# The Dynamic Entropy of Tumor Immune Infiltrates: The Impact of Recirculation, Antigen-Specific Interactions, and Retention on T Cells in Tumors

**DOI:** 10.3389/fonc.2021.653625

**Published:** 2021-04-22

**Authors:** Tiffany C. Blair, Alejandro F. Alice, Lauren Zebertavage, Marka R. Crittenden, Michael J. Gough

**Affiliations:** ^1^ Molecular Microbiology and Immunology, Oregon Health and Sciences University (OHSU), Portland, OR, United States; ^2^ Earle A. Chiles Research Institute, Providence Cancer Institute, Providence Portland Medical Center, Portland, OR, United States; ^3^ The Oregon Clinic, Portland, OR, United States

**Keywords:** T cell, recirculation, tumor, clonality analysis, retention, recruitment

## Abstract

Analysis of tumor infiltration using conventional methods reveals a snapshot view of lymphocyte interactions with the tumor environment. However, lymphocytes have the unique capacity for continued recirculation, exploring varied tissues for the presence of cognate antigens according to inflammatory triggers and chemokine gradients. We discuss the role of the inflammatory and cellular makeup of the tumor environment, as well as antigen expressed by cancer cells or cross-presented by stromal antigen presenting cells, on recirculation kinetics of T cells. We aim to discuss how current cancer therapies may manipulate lymphocyte recirculation versus retention to impact lymphocyte exclusion in the tumor.

## Introduction

The evolutionary development of an adaptive immune system requires different systems to localize responses. An adaptive immune response against a specific virus can result in viral antigen-specific B cells and T cells that remain throughout life (1). The progressive accumulation of such responses over the lifetime of an individual would make it impractical to have all of the different types of antigen-specific cells located at all possible sites of infection. Rather than distribute all immune cells uniformly, the mammalian immune system uses a system of sensors and rapid responses to mobilize responses to a location. Compared to a fully distributed immune system this will result in a delay, but allows a more flexible system.

To achieve this the mammalian immune system employs selective sensors, which provide an initial indication of the type of immune response required, to mobilize suitable cells. For example, intracellular sensors for nucleic acids that might detect a viral infection, such as TLR3, RIG-I-like receptors and cGAS/STING can generate a different pattern of cytokine responses to extracellular sensors for bacterial components such as TLR2 and TLR4 (2, 3). Moreover, the various sensors are not uniformly shared across innate immune cell types, so that specific immune cells can specialize in detecting certain infections (3, 4). In this way the immune response is pre-warned of the nature of the infection, and the appropriate adaptive response that is needed.

Among the cells that participate in the adaptive immune response, T cells have a particular limitation in that they must be in physical proximity to a target cell expressing antigens in order to exert their effect. B cells can retreat to stable niches in the bone marrow and secrete antibody for the duration of the animal’s lifespan (1). This isn’t possible for T cells. To overcome this limitation, the immune system exploits a mechanism to move antigens to T cells, and T cells to antigen (5). Naïve T cells do not travel to tissue sites and so cannot scan for their cognate antigen at the site of infection. Instead, dendritic cells are able to carry antigen from tissues to draining lymph nodes where they are able to present the antigen to naïve T cells and initiate antigen-specific immune responses. Mathematical modeling suggests direct lymph node entry of T cells is low in the absence of inflammation (6). For naïve T cells, entry is classically dictated by CCR7, which also directs T cells to the vicinity of dendritic cells within the T cell zone of the lymph node (7). However, inflammation in the upstream site can lead to remodeling of the lymph node to increase infiltration of naïve T cells and recruitment of all T cells to the node (7, 8). Once in the lymph node, T cells that continue to receive chemokine signals, or are held in place *via* cognate interaction and retention signals such as CD69, will remain in the lymph node. However, there is an ongoing pull *via* S1PR1 on the T cells and S1P in the lymphatics that results in exit of T cells that fail to meet their cognate ligand or have disengaged from antigen presenting cells (9). This ongoing pressure to leave ensures continued recirculation of T cells in search of cognate ligands.

Once they are antigen experienced, T cells are subsequently able to travel through the blood to tissues due to a range of changes including altered selectin expression (7) and explore local MHC for their cognate antigens. Importantly, a recirculation system exists to return these T cells through the draining lymphatics and back into blood circulation (5, 7). Without such a system of recirculation, antigen-experienced cells would be ‘lost’ to the tissues resulting in a progressive loss of antigen-experienced cells from the circulation. This is the critical feature that provides our circulating, distributed form of adaptive immune system. While the principle of recirculation is well known and a fundamental of basic immunology, what is often unappreciated is the rate of recirculation. In studies performed over 50 years ago it was demonstrated that the total blood pool of lymphocytes can be refreshed 11 times per day based purely on the output from the thoracic duct (10). The drug FTY720, which prevents lymphocyte egress from lymph nodes by blocking Sphingosine-1-phosphate receptor 1 (S1PR1), can result in complete loss of thoracic duct lymphocytes within 4 hours (11) and 90% loss of peripheral blood lymphocytes in 3-24 hours (12), demonstrating an extremely high recirculation rate. While FTY720 treatment rapidly removes lymphocytes from the blood it has a lesser effect on the tissues, and these data have allowed investigators to calculate transit times of approximately 18 hours through tissue parenchyma (13). Thus, T cells spend their least amount of time in the peripheral blood, with estimates ranging from 1-2 circulations through the heart, which corresponds to timescales of minutes in the bloodstream of mice (6, 14). By contrast lymph node transit even in the absence of cognate antigen can take approximately 10 hours (6, 15). In models of long-term memory to viral infection, it was found that less than 4% of the virus-specific T cell memory population was present in the peripheral blood at any one time (16). Thus, T cells spend the majority of their time in tissues or secondary lymphoid organs scanning for cognate antigens.

As with all rules, there are exceptions. Recently, resident memory T cells (Trm) have been described that remain in peripheral sites long term and provide rapid local antigen-specific responses (7, 16, 17). The majority of experiments that identified these cells were performed in very clean laboratory settings where the mice had an extremely limited history of infection. This makes it difficult to assess how diverse the Trm pool is in peripheral tissues, since there will clearly be a ‘space’ constraint in supporting a fully diverse T cell population at all peripheral sites. Human neonates have increased populations of naïve T cells compared to adult humans, and antigen-experienced populations are less frequent in neonatal tissue where there is likely a limited experience of antigen (18, 19). Therefore, with antigenic experience, peripheral niches are populated with antigen-experienced cells. By analyzing wild and pet-shop mice, Beura et al. demonstrated a dramatically higher population of memory T cells populating peripheral tissues of wild mice compared to laboratory mice (20). In normal human pancreas tissue, Trm were found to express similar defining markers such as CD69 and CD103, but were phenotypically distinct from jejunal Trm (21). This included decreased expression of a range of inflammatory markers (21), which may relate to the lower ongoing exposure to infectious agents in the pancreas. As would be expected given the potential for differing local antigen exposure, Trm clones in the pancreas and jejunum also had limited overlap (21). In agreement with this, Trm in normal lung but not other sites demonstrated reactivity to influenza antigens (22); however, influenza-specific Trm in the lung share clonotypes with non-Trm memory subtypes in the lung (23). These data demonstrate that while Trm provide local recognition, their function is reinforced by recirculating populations. Difficulties in comparing clonotypes is highlighted by Schoettler et al, who demonstrated using lung samples that only approximately 5% of more than 100,000 TCR clones were found in more than one tissue or patient sample and only TCRs expressed by CD4 T cells were identified as shared across multiple memory populations in both the lung and lung-draining lymph nodes (24). It remains unclear quite how comprehensively protective the Trm cell response can be given the limited size of the Trm niche in any one place, compared to the diversity of the combined repertoire that is recirculating or resident elsewhere at any moment. It is possible that the Trm niche reflects recent antigen exposures, and pre-existing cells are displaced to new recruits. In this way the peripheral resident population will proportionally represent frequent infectious agents, including non-pathogenic colonizing organisms and this resident response will therefore be of most use for rapid responses to these most frequently exposed agents. This is supported by data in lung infection, where repeated antigen exposure ensures a durable lung Trm population (25). In this, way, earlier exposures to a common organism may come to dominate the local response. By monitoring the response to viral infections containing model antigens, Muschaweckh et al. demonstrated a selective preference for T cells specific for the model antigen Ovalbumin to form Trm as compared to T cells specific for the viral antigen B8R_20_ (26). However, if the tissue was first allowed to form Trm specific for the viral antigen B8R_20_ through prior infection, then B8R_20_ -specific T cells dominated the Trm niche even after challenge with a B8R_20_ and Ova-expressing virus (26). These data suggest that Trm formation is affected by immunodominance (discussed more later), and that existing Trm populations can outcompete simultaneous incoming new responses to retain their place in the tissue niche. Importantly, T cells specific for the same antigen can be found as Trm and as classical circulating memory populations (27), so even if Trm that recognize a specific infection are lost from the tissue niche through progressive rounds of infection with other agents, the circulating memory can remain.

The principle of recirculation is also essential to overcome the fact that T cell recruitment to tissue sites is not antigen specific. Recruitment to peripheral sites is dependent on inflammatory patterns. Inflammation in the tissue site generates cytokines such as TNFa, which activates endothelial cells to express adhesion molecules such as ICAM1 [reviewed in (28, 29)]. This is critical to initiate the process of tissue entry by lymphocytes by allowing rolling along the endothelial surface of the blood vessel lumen. In addition, local inflammation results in chemokine secretion, and chemokine binding to receptors on rolling cells permits changes in adhesion to tight binding, and eventually diapedesis through the endothelia and into the tissue (28). This means that an infection that results in a local inflammatory response triggered *via* infection sensors will non-specifically recruit any T cells expressing selectins and appropriate chemokine receptors, regardless of TCR specificity. Since the recruited T cells need both the correct chemokine receptors and activation-regulated adhesion molecules to permit diapedesis through the vasculature into the tissue, there will be selection for activated T cells (30). In animal models, there is likely only one major ongoing infection at any one time, so the majority of the emerging activated cells are likely specific for the infection (20). However, in a human there are likely multiple ongoing infections simultaneously occurring at different sites, therefore T cells specific for an ongoing flu infection may also be recruited to the site of an infected splinter, and vice versa. Recirculation permits non-specific cells to leave the tissue and be available for recruitment again. This can also result in dominance of a highly inflamed tissue. For example, a lung infection can recruit T cells specific for other pathogens to the lung as part of the local inflammatory response (31). Similarly, in tumors, T cell recruitment is not antigen-directed but instead attracts all T cells with appropriate activation markers (32). To return these cells to the general circulation, and ensure they are available to respond to their cognate targets should they return, requires efficient recirculation.

Using tools such as Kaede mice, where cells can be photoconverted at a specific site then followed for their movement (33), it is clear that not all T cells are equivalent in their recirculation dynamics. By labeling the tumor then analyzing the tumor draining lymph node, within 1 day of tumor labeling the majority of emigrated cells are dendritic cells and T cells (34). Lymph nodes draining tumors exhibited a much higher overall number of recirculating cells than normal skin (34), suggesting a high rate of immunosurveillance in tumors, despite tumor progression. Though the number of T cells in the lymph node that had been in the tumor at conversion peaked at 1 day following conversion, tumor-originating cells were still in the lymph node at day 3 (34). It is not clear whether this is continued emigration or retention of these cells in the lymph node, but the tumor was still observed to hold a large proportion of the converted T cells at day 3 after conversion (34). As would be expected based on the requirements for initial tumor infiltration, the majority of cells recirculating to the draining lymph node are enriched for effector and central memory phenotypes. Using the Kaede system to convert skin resident T cells in infectious models, Park et al. demonstrated that Trm in the skin remain in place following viral rechallenge, and do not recirculate *via* the draining lymph node (35). However, circulating virus-specific T cells are recruited to the skin site, and themselves become Trm following infection (35). In tumor models, by day 3 following conversion of Kaede cells in the tumor, some of the converted T cells are detectable in the lymph nodes draining an identical tumor at a distant site but the converted cells are poorly detectable in distant lymph nodes that do not drain tumors (34). These data suggest that by this time point dissemination through the peripheral blood has occurred and antigen-mediated retention has allowed accumulation of tumor-specific populations in distant lymph nodes. Thus, even following exit from tumors, recirculation and accumulation at sites of distant antigen are rapid *in vivo*.

In this review we will discuss how the principle of T cell recirculation impacts lymphocyte exclusion in the tumor environment. In addition, we will explore the effect of therapy on lymphocyte numbers in the tumor, with a focus on the differing effects on recruitment versus retention. In response to cognate antigens T cells can also proliferate locally, which will also result in T cell accumulation at the tumor. In our review we will not discriminate the mechanisms that result in lymphocyte arrest versus proliferation in response to cognate antigen. Together these are grouped as retention mechanisms, rather than recruitment mechanisms. Different treatments may differently affect recruitment versus retention of lymphocytes, and this may play a role in their successes and failures.

### Lymphocyte Recirculation Kinetics

**Figure 1:**
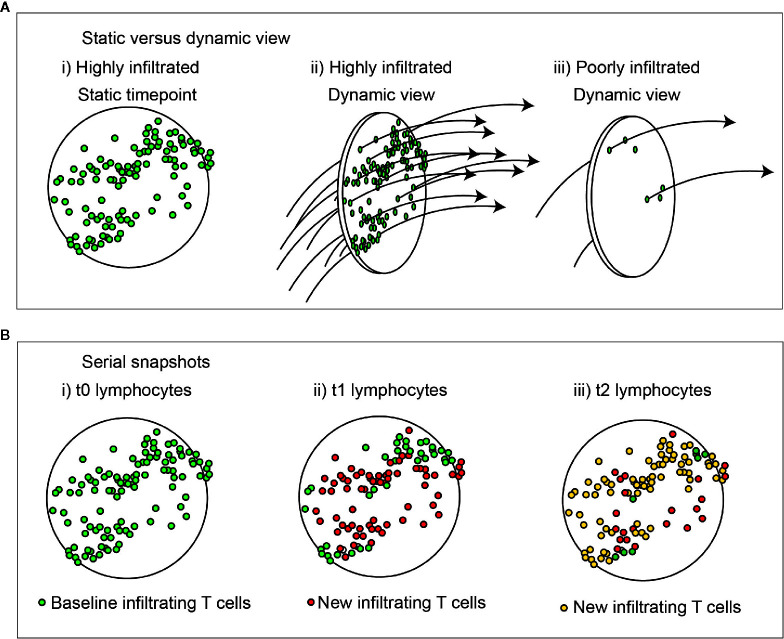
Dynamic view of cell infiltration in tumors. **(A)** A highly infiltrated tumor may also represent a high rate of throughput of immune cells. i) a static view shows large numbers of T cells in the tumor. ii) a dynamic view shows a high rate of surveillance and recirculation. Iii) a dynamic view of a poorly infiltrated tumor shows a low rate of surveillance and recirculation. Understanding the kinetic will help understand the rate of accrual versus accumulation in the environment. **(B)** Change in lymphocytes in tumors over time with unchanged overall infiltration. i) Lymphocytes present at baseline are green. ii) Those newly present at the second timepoint are red, and iii) newly present at the third timepoint are yellow. At each timepoint not all cells are replaced, and those exhibiting prolonged interactions in the tumor are more likely to have engaged their cognate antigen.

Most assessments of tumor infiltration view only a single snapshot in time within the tumor. Although such assessments may show a high degree of tumor infiltration by T cells, it may also represent a tumor with a high throughput of T cells entering and leaving the tumor, so actually representing a high rate of surveillance (**Figure 1A**). If we consider the lymphocytes in a tumor over time, we can anticipate that some continue to recirculate, while others remain (**Figure 1B**). Assuming constant inflammatory conditions there is little reason for the overall number of cells to change over time, but when comparing the specificity of the T cells present at any two timepoints we could expect completely different T cells are infiltrating the tumor. With inflammatory flux, the numbers may go up or down as recruitment changes, but their overall time spent in the tumor may be unaffected if their retention is unchanged. Tools such as TCRSeq allow us to sequence the TCR of T cells and examine their diversity (36, 37). Expansions of specific T cells as clonal populations are detectable as repeated TCR sequences with increased frequency and using this technique to examine tumors shows that there are measurable clonal expansions in tumors. If we were to use TCRSeq to compare TCR clones in the tumor over time, we would anticipate an overall change in TCR clonotypes according to the degree of recirculation. Among the population that is retained over time, we would anticipate enrichment for properties of resident cells, such as Trm, or ongoing antigen engagement that results in prolonged adhesive interactions with their targets that impacts retention. This is supported by data from patient tumors, demonstrating that tumor reactive CD8 T cells in tumors express markers associated with tissue residency (38, 39). Multiple additional markers that may define the Trm phenotype have been described (40); however, experimental limitations means that residency has only been proven in murine systems. Nevertheless, ongoing studies in human tissues have identified shared features of cells expressing the canonical CD103/CD69 signature in humans (40). Bystander viral-specific CD8 T cells in tumors can also express these Trm markers (39), therefore CD39, a marker of chronic T cell activation is useful in distinguishing between the bystander and tumor-reactive T cell populations (38, 39). Duhen et al. used the combination of CD39 and the Trm marker CD103 to enrich for tumor-reactive CD8 T cells, and compared these cells in the tumor to those from the blood and lymph nodes (38). The CD103^+^CD39^+^ (double positive – DP) CD8 T cells in the tumor were shown to have clear enrichment for specific clonotypes. Duhen et al. demonstrated that the greatest TCR diversity was found in CD8 T cells in the blood, and the lowest TCR diversity was found in the DP CD8 T cells in the tumor (38). DP CD8 T cells did not significantly share TCR sequences with CD8 T cells in the tumor draining lymph node or the peripheral blood, while CD103^-^CD39^-^ (double negative – DN) CD8 T cells did share TCR sequences with circulating cells (38), suggesting that the DP CD8 T cells are selectively retained within the tumor and the DN CD8 T cells are recirculating. This also means that examining any two different time-points might show a similar proportion of clonally expanded cells, but the non-specific clones would change over time and the cells that are present at both time points would be expected to be enriched for tumor antigen-specific cells. This proposition appears to be supported by current data. Using a combination of scRNASeq and TCRSeq Yost et al. demonstrated clonal expansions of CD8 T cells in tumors were enriched for exhaustion markers (41), and that these same cells also exhibited evidence of tumor reactivity based on expression of CD39 and CD103. Importantly, they found little overlap in TCR clonotypes between the exhausted population and CD8 T cells with effector phenotypes, suggesting that these cells have distinct specificities (41).

While we assume that recirculation will result in selective replacement of only non-specific cells, and that while the overall diversity of the infiltrate might remain consistent, there will be some notable caveats. We all have clonally expanded T cells specific for common viruses meaning that our circulating T cell pool is not uniformly distributed among possible TCR sequences. These clonally expanded T cells would also be present in the tumor through non-specific recruitment, but these would not be expected to be selectively retained. However, any chronically active T cell populations, such as those for CMV or EBV might be enriched in the activated T cell pool that is recruited to tumors. Thus, not all clonally expanded cells in tumors can be predicted to be tumor specific. Scheper et al. cloned the TCR from tumor infiltrating T cells and evaluated their specificity for autologous cancer cells. They found that only a small proportion – from 1-10% of T cells in the tumor were specific for cancer cells, and that in some examples T cells specific for EBV were three times more frequent in the tumor (42). As many as 3% of CD8 T cells infiltrating tumors have been shown to be specific for a CMV epitope (39). This number likely varies significantly between tumors, according to the degree of non-specific recruitment and the antigen-specific retention. For example, in the above paper, the authors analyzed a melanoma specimen where 90% of the tumor-infiltrating T cells expressed PD1, and 50-80% of all T cells were estimated to be tumor specific (42). Shitaoka et al. demonstrated that CD8^+^CD137^+^ cells represented 10-70% of CD8 T cells infiltrating human tumors, and a large proportion of these were clonally expanded (43). In murine tumors CD8^+^CD137^+^ cells represented up to 5% of tumor-infiltrating CD8 T cells, and TCRs from clonally expanded CD8^+^CD137^+^ cells were mostly tumor-reactive (43), suggesting that this population also enriches for tumor-reactive cells. Tumor-specific T cells generated *ex vivo* and adoptively transferred into an animal circulate widely, with no particular selectivity for a tumor expressing the cognate antigen (44). However, functional activation was limited to the site of antigen. These data suggest that non-tumor specific clonally expanded populations would be expected in the tumor, and tumor-specific clonally-expanded populations would be expected elsewhere. In this way clonal expansion may be an insufficient measure of specificity for antigens at that site. This is supported by current data. Penter et al. explored clonal expansions in colorectal tumors as well as uninvolved sites, and found similar rates of clonal expansions inside and outside the tumor (45), indicating that clonal enrichment is not unique to tumors. However, while on aggregate uninvolved and tumor regions had similar expansions, the data suggest that individual patients may exhibit enrichment for clonal expansions in the tumor. The tumor-associated clones exhibited increased expression of activation and exhaustion markers such as PD1, which are not seen in non-tumor clones (45), suggesting they are chronically recognizing antigen while in the tumor. Clonally expanded populations in the peripheral blood were stable over time (45), likely representing circulating memory populations specific for common targets such as EBV and CMV, and importantly a dominant CMV-specific clone was shown to be expanded in the blood, tumor, and uninvolved tissue site, demonstrating that these cells recirculate widely. In addition, clones that were highly expanded in the tumor were also detectable in the uninvolved tissue site (45), suggesting that tumor-specific cells may also be recirculating or can take up residency elsewhere. Analysis of lung tumors versus distant normal lung tissue demonstrated that highly expanded clones were more frequent in normal tissue than the tumor and the T cell in the tumor had greater TCR diversity (46), suggesting that the tumor recruits more non-specific T cells compared to normal tissue. Interestingly, in this and other studies there are data suggesting that a lower clonality and an increased T cell diversity in tumors is associated with worse outcome to conventional therapies and immunotherapies (46, 47). This would fit with clonal populations representing accumulated tumor-specific T cells among a background of diverse non-specific T cells. In this way, overall infiltrate is less informative than infiltrates of specific T cells.

**Figure 2:**
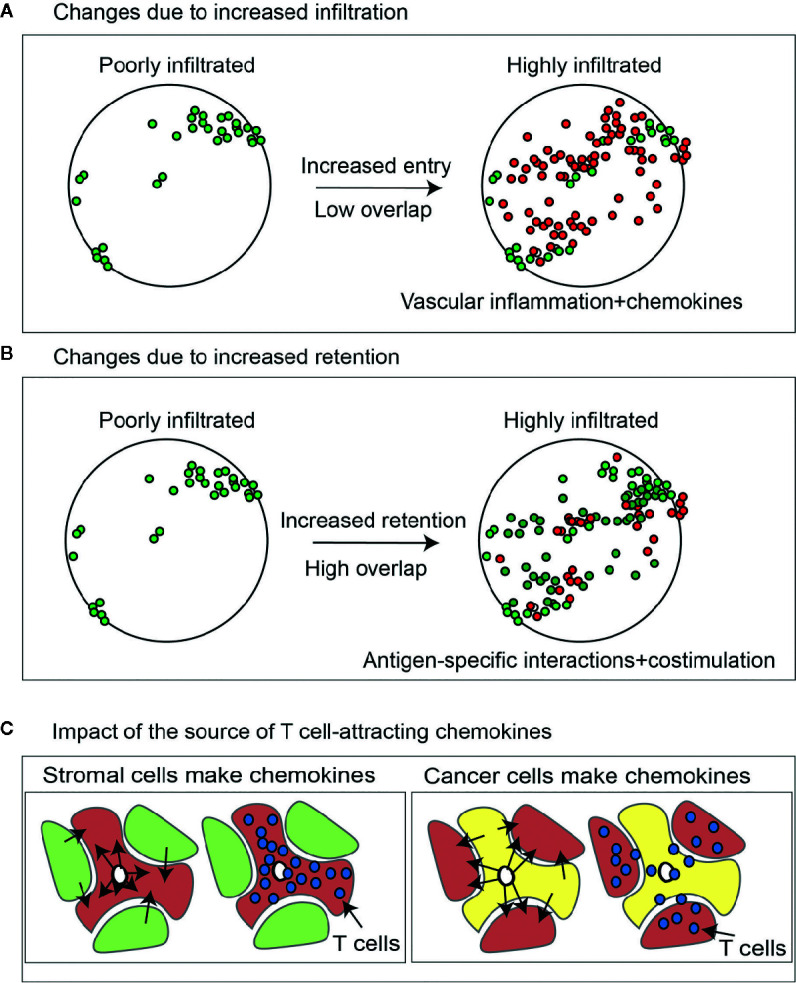
Change in lymphocytes in tumors due to increase infiltration versus increased retention. **(A)** Effect of increasing lymphocyte infiltration *via* chemokines and inflammatory changes in the tumor vasculature on T cell diversity in the tumor. Lymphocytes present at baseline are green. Those newly present at the second timepoint are red. **(B)** As with **(A)**, but the effect of increasing T cell retention *via* increased antigen-specific interactions *via* altered antigen presentation or costimulation. **(C)** Higher expression of chemokines that can attract activated T cells in cancer cells will cause proportional enrichment of immune cells in the vicinity of cancer cell nests. By contrast higher expression of the same chemokines in the tumor stroma may cause their enrichment in the stroma but exclusion from the cancer cell nests, limiting their cytotoxic potential.

For these reasons, a critical measure of tumor specificity or selectivity may be serial assessment. However, such analyses are rare. In part this is due to the clinical scenarios, since most analyses are performed on single biopsies or a tumor resection specimen. Yost et al. used bulk TCRSeq to compare T cell clonality in untreated tumors at two timepoints and found no significant changes in the TCR sequences present in clonally expanded populations over time (41). This was in contrast to tumors sampled before and after PD1 blockade, which resulted in an influx of new expanded clones (41). Later we will explore the effects of therapy on recirculation kinetics, but it is reasonable that at baseline the T cell infiltration of any tumor will be directly related to recruitment and retention, and be generally split into rapidly recirculating non-specific cells and selective retention of antigen-specific cells. Thus, increasing recruitment *via* inflammation and chemokines has the potential to increase the diversity of T cells in the tumor, but this occurs without any selectivity for tumor-specific cells (**Figure 2A**). As we will discuss later, recruitment is not selective for tumor-specific T cells, and will attract any cells expressing the appropriate chemokine receptors, whether tumor-specific or CMV-specific. By contrast increasing antigen presentation and altering recognition thresholds using costimulatory agonists or coinhibitory blockade has the potential to increase clonality by increasing retention of tumor-specific cells (**Figure 2B**).

Intravital 2 photon microscopy has helped understand the dynamics of T cells in tumors. Using cancer cells expressing ovalbumin as a model antigen to view tumor-specific responses of OT1 TCR transgenic T cells, Breart et al. demonstrated that in a highly responsive model, transferred T cells were first visualized in the vicinity of vascular entry points and rapidly spread throughout the tumor resulting in cure (48). Salmon et al. demonstrated higher motility of T cells in the tumor stroma, and their comparative exclusion from tumor nests (49). Motility was highest in the immediate perivascular location, with fibers of the extracellular matrix serving to prevent direct T cell interactions with cancer cells (49). Initial estimates suggested that tumor-specific T cells and cancer cells required a 6hr duration of interaction to result in cancer cell death (48). This timing is interesting since only a proportion of T cells exhibit a longer duration interaction with cancer cells in tumors, with the majority exhibiting short-term interactions with multiple cells despite having specificity for tumor-associated antigens (50). Random migration of the T cells occurs prior to stable interaction with the target, and the kinetics of T cell movement differs depending on the presence of the cognate antigen (50). While T cells pause when meeting cancer cells that they can recognize, in models where the T cells are known to be responsible for curing tumors, they resume their motility once they successfully kill their targets (51). Interestingly in these models, T cells could be seen actively moving along the exterior of blood vessels in the periphery of the tumor, which would represent a stromal location, and they adopted a non-motile appearance once detached from the blood vessel (51). T cells only entered deeper regions of the tumor when cognate antigen was present, and these changes took more time and followed the kinetic of successful tumor elimination at the periphery. This potentially relates to inflammatory changes in the vicinity of T cells actively engaging antigen that are propagated to neighboring regions that were formerly poorly inflamed and thus poorly infiltrated, or due to the T cells being restrained in the periphery until cancer cells were eliminated and they could resume their motility.

Studies using pertussis toxin demonstrate that chemokine signals are necessary for T cell motility within the tumor stroma (49), and cancer cells engineered to express chemokines can increase recruitment of T cells into the stroma (52), and on into cancer nests (49). In such a scenario, the ability of newly recruited T cells to meet their cognate antigen presented on cancer cells will be highly dependent on local chemokine signals that recruit T cells out of the stroma and towards cancer cell nests (**Figure 2C**). At the same time, inflamed lymphatic endothelial cells may secrete their own chemokines (53) and direct T cells out of the stroma to recirculation. The pre-existing Trm populations resident within cancer cell nests have both a phenotype that encourages adhesion to the cancer cells (38, 54) and lack SIP1R resulting in limited capacity for lymphatic traffic (55). Thus, once past the tumor stroma circumstances favor tumor residency versus recirculation of tumor antigen specific T cells.

### Chemokine Modification of Tumors to Increase Recruitment

To understand recruitment and retention it is useful to take the example of two different tumors, one highly infiltrated and another poorly infiltrated with T cells. At baseline, we have no information as to whether the tumors are highly infiltrated due to an increased recruitment of T cells to the tumor, or increased retention of T cells within the tumor (**Figure 2**). If recruitment is the key criteria, then manipulating inflammatory signals within the tumor will influence infiltration. Over the last few decades, we and others have explored modification of cancer cells with cytokines and chemokines to increase immune cell infiltrates into tumors. Engineering tumors to express chemokines that attract T cells results in increased T cell infiltration and increased tumor immunogenicity (56–58). These data suggest that T cell recruitment to tumors is limited by suboptimal chemokine expression. Analysis of tumors with high versus low T cell infiltrates demonstrated that expression of a panel of 12 chemokine genes could predict increased T cell infiltration into tumors (59, 60). However, in view of the recirculation behavior of T cells, how will increasing chemokine levels change anti-tumor immunity? As discussed above, increased inflammation in the tumor or increases in chemokine expression will lead to a non-specific influx of T cells into these inflammatory areas, where the majority of cells are not tumor-specific. Among these recruited T cells there may also be populations of unconventional T cells, for example gamma delta T cells. Such cells can share recruitment mechanisms, and while they may not recognize tumor antigens, they may respond to the altered metabolic status in the tumor environment (61).

The fact that tumors may progress despite the presence of targetable neoantigens can in part be explained by immunological ignorance (62). T cells specific for cancer cells can recirculate through the tumor without impacting the tumor growth, unless given some external stimulus. This can be due to limited ongoing cross presentation (63, 64), or limited direct presentation due to low expression of the cognate antigen or low levels of antigen processing and presentation in the cancer cells (65, 66). In some circumstances these limitations can be overcome through increased antigen release from the cancer cells (67, 68). However, T cell ignorance of tumors can also be impacted by a poor rate of recirculation through tumors as well as their limited exposure to tumor-associated antigens. In addition, whether T cells are ignorant of the tumor or are actively recirculating through the tumor, these T cells will still face the broad range of immune suppression mechanisms that operate in the tumor environment. These issues are well reviewed (69–71), and such immune suppression may be the dominant pathway regulating adaptive immune control of growing tumors.

If chemokine levels in a tumor alter over time, the proportion of newly recruited cells that are tumor-specific before and after chemokine expression would be anticipated to remain identical, though their absolute numbers would be expected to change. However, increasing chemokine levels may increase the rate of recirculation, and therefore increase the likelihood that a tumor-specific T cell can meet it’s cognate ligand. In this way, increased T cell recruitment may result in an increased proportion of tumor-specific T cells in the tumor through a more efficient screening of the recirculating T cell repertoire. For this to impact tumor control, entry to the tumor must have been the limiting factor preventing tumor-specific T cells from exerting their function. This is plausible, since as described above, low chemokine expressing tumors and low T cell infiltrated tumors have worse outcome than their matched counterparts (72–76). CXCR6 has been shown to play a role in the recruitment of Trm to tissue sites and their retention in tissues *via* its ligand CXCL16 (77). However, this chemokine receptor and ligand are not tissue specific. Up to 20% of all peripheral blood CD8^+^ T cells express CXCR6 in cancer patients (78) and healthy donors, and CXCR6-mediated recruitment occurs in multiple healthy tissues including the lung and liver (77, 79). The percentage of CXCR6+ cells in the peripheral blood is much lower in mice with no history of infection, but is upregulated following infection (80). Therefore, CXCR6-expressing T cells may represent the diverse array of immune responses occurring in humans. Consistent with this, from 20-60% of EBV-specific T cells circulating in patients express CXCR6, and CXCR6 can be rapidly upregulated on antigen rechallenge (78). Importantly, CXCL16, the ligand for CXCR6, is upregulated in normal tissues following infection (79, 81) so all CXCR6-expressing T cells may be recruited to the site along with specific T cells. CXCR6 is enriched on cells that infiltrate tumors, as are other chemokines associated with activated T cells, such as CCR5 (78). In particular, CXCR6 is particularly associated with a Trm population (77, 82, 83). Given the low recirculation of Trm phenotype cells and the high proportion of circulating CD8 T cells that express CXCR6, it is unlikely that CXCR6 is a specific means to recruit Trm. Alternatively, since CXCR6 is an activation marker on T cells, it is potentially a marker of T cells that have received additional cognate stimulation in the tumor environment, and may serve to retain antigen specific cells where both CXCL16 and the cognate antigen co-exist. In this way chemokine receptors that are induced by activation are likely to be enriched on the antigen-specific populations in the tumor environment, akin to the activation markers CD69 and CD39. It may also be important that CXCL16 is an unusual chemokine in that it is membrane bound, until cleaved by proteases that are regulated under inflammatory conditions and during cancer treatment (84–87). Therefore, CXCR6 may generate a retentive niche for antigen-reactive cells in close contact with epithelial cells, or recruitment from systemic circulation under inflammatory conditions.

Where chemokine expression is already high, or T cell infiltration is high, it would seem that recruitment is not a limiting factor in tumor control. It is logical that a tumor that has abundant T cells yet continues to grow may be more impacted by other issues (88). It is possible that these infiltrating T cells are in an unsuitable location, are suppressed, are unable to engage with antigen presented by cancer cells, or are simply not specific for the cancer cells. In addition, as will be discussed below, chemokine expression by the cancer cells might additionally impact the distribution of T cells within the tumor environment, encouraging T cell migration through the stroma and to cancer cells nests. For this reason, experiments that evaluate the role of chemokine expression on immune responses artificially alter the biology of the system. When cancer cells are engineered to express chemokines, as we have used in the past (52, 56), the chemotactic gradient will peak around the cancer cells, so recruitment of immune cells will be to the cancer cell nests. By contrast increased chemokine expression by cells of the tumor stroma may lead to a non-productive accumulation of T cells in the stroma without impacting their contact with cancer cells. Non-cancer cells of the tumor stroma are critical sources of chemokines in tumors, and altering the recruitment of T cells into versus out of the stroma can have therapeutic consequences that do not relate to the overall chemokine production in the broader tumor environment (**Figure 2C**). A heightened inflammatory environment that is restricted to the stroma may therefore negatively impact functional tumor control. Moreover, the infiltrating non-specific T cells may limit the ability of the specific cells to establish a niche and engage cognate antigen. This phenomenon is evident in a model of diabetes, where increased infiltration of non-specific T cells into the islet actually reduced the ability of islet-specific T cells to cause autoimmune diabetes (89). In this model, there is a threshold number of islet specific T cells that are necessary to bring about diabetes (90); however these islet-specific T cells represent a small proportion of the T cells infiltrating the islet (90). By providing large numbers of T cells of irrelevant specificity through a vaccination approach, the islet-specific population in the islet were less activated and less effective (89). This data is likely very impactful to cancer in patients, where ongoing irrelevant immune responses are likely present to a much greater degree than are present in murine models in clean animal facilities (91), and any attempt to increase recruitment to the tumor will occur regardless of specificity. In this scenario, the mechanisms impacting retention and activation may be more critical than recruitment to improve anti-tumor immunity.

### Role of Antigen in Tissue Retention

Advances in genomic analysis of tumors and bioinformatic models to identify tumor mutations has resulted in a dramatic increase in the discovery of patient-specific neoantigens (92). The number of these antigens per patient vary considerably, but thus far most patients tested have been found to have targetable neoantigens and T cells that can recognize them (93–95). However, when considering the additional restriction based on MHC-binding of any neoantigens, it is theoretically possible that a poorly mutated tumor could have no targetable tumor neoantigens. This may be a stronger possibility in pediatric malignancies, where highly penetrant driver translocations/mutations can result in tumorigenesis with few additional passenger mutations (96–98). In these cases, recruited T cells would have no interaction with cognate targets, and would freely recirculate with no possible retention of specific cells. However, since recruitment is not antigen specific, it is possible that such a tumor could still have T cell infiltrates. Spranger et al. examined the neoantigen profile of tumors that were highly or poorly infiltrated with T cells, and found no correlation between the number of antigenic targets and the numbers of T cells infiltrating the tumor (99). These data suggest that T cell infiltration is unrelated to antigen density. However, in addition to neoantigens, there are an array of tumor-associated antigens (100) and in some cases viral antigens that can be effective targets for T cells. For example, in a recent clinical study in head and neck cancer, CD8^+^ T cells in the tumor did not make measurable responses to any of the mutated neoantigens that were present in the cancer cells (101). However, the T cells made strong responses to the E6 and E7 proteins from human papilloma virus. Notably, these responses were exclusively found in the CD103^+^CD39^+^ population of resident CD8 T cells (101), demonstrating that these cells are not restricted to mutated neoantigen reactivity. In B16 tumors in murine models, approximately half of the clonally expanded T cells in the tumor were reactive to the unmodified gp70 epitope that is shared in many murine tumors (43). Thus, mutated neoantigens may not be essential for adaptive immune control of tumors.

In interpreting antigen density we must be cautious not to assume that we will make T cells specific for all potential targets. In infectious disease models, despite a wide range of potential antigenic targets the immune response generally focuses on a small number of antigens. This is known as immunodominance and occurs in antibody and T cell responses (102–104). This suggests that once a tumor passes some antigenic threshold, immunodominant antigens may focus the immune response around a restricted set of T cells, and additional antigens may not impact the T cell response. Immunodominance is a potential problem in the immune response to infectious agents and to cancer, since the high antigen specificity can generate selective pressure that can permit outgrowth of variants. This has been observed as antigenic drift in infection (102) and immunoediting in tumors (105). Thus, despite large numbers of potential neoantigen targets in patient tumors, commonly only a very small number of tumor-specific clones can be cultured, often as low as 1-2 clones per tumor (92, 95). While this may be a technical issue relating to T cell expansion from tumor tissue given their suppressed status, if immunodominance limits the number of responses per patient it may provide an alternative explanation for the disconnect between the number of neoantigens and the degree of T cell infiltrate. However, this is also an opportunity for therapy, since we have the potential to introduce additional T cell responses capable of contributing to tumor control. Linnette et al. identified neoantigens present in tumors using genomic sequencing, and identified T cell specific for these tumor neoantigens using peptide stimulation and a DC vaccination approach in patients (106). Prior to vaccination, T cells specific for tumor antigens were below detection limits in the peripheral blood, but T cells specific for neoantigens could be expanded from blood and tumor *ex vivo*, with more of them found in the tumor (106). Vaccination resulted in expansions in neoantigen-specific T cells in the peripheral blood, but importantly, these experiments allowed the investigators to identify neoantigen-specific TCR sequences. TCR sequencing of the tumors demonstrated that the majority of these tumor-specific TCR sequences were absent from the tumor prior to vaccination, and even where present only a proportion of the potential specificities were detectable (106). Thus, patient tumors exhibit only a small proportion of the potential reactivity to unique neoantigens, supporting both some degree of immune ignorance and some degree of immunodominance in tumors. Kalaora et al. comprehensively characterized potential neoantigen targets in melanoma patients and corresponding TCR sequences in T cells expanded *in vitro* from tumors (107). Importantly, while there was significant variability between patients, distinct metastases within an individual patient overlapped in both neoantigen targets and TCR sequences present. In one example, these experiments demonstrated that 11 TCR sequences accounted for 90-99% of the tumor specificity (107). Zhang et al. demonstrated that the degree of clonality in tumors was positively correlated to the overall mutational burden (108). Interestingly, this paper also demonstrated a negative correlation between clonality and the percent tumor in the specimen (108), suggesting that an increased stromal component results in a decreased clonality likely due to increased infiltration of non-specific T cells in the stroma. Recent studies have demonstrated that a ‘mutator phenotype’ associated with loss of mismatch repair pathways is a stronger predictor of outcome than quantity of mutations (109, 110). Such tumors have increased T cell infiltrates counterbalanced by local immune suppression, including increased PDL1 expression (109, 111–113). However, it is unclear whether the mutations in these tumors dictates this infiltration phenotype. Tumors with the mutator phenotype have a more rapid tumorigenesis (114) and these tumors are highly inflamed before they have a high mutational burden (115). It is plausible that the mutator phenotype regulates immune activation through multiple mechanisms of which increased mutational frequency is only one (116).

One significant feature of tumors that is different from acute models of infectious disease is that the antigen target is chronically present in tumors. In most acute infectious disease models, antigen is present for only a few days to weeks in the infection site, and cross-presented antigen has a similarly short half-life. Following elimination of the pathogen, antigen is lost from the environment and as discussed above, the antigen-specific T cells remain as both tissue infiltrating resident memory T cells and circulating conventional memory T cells (17). Broadly, once established these are thought to be non-overlapping populations in the absence of further antigen exposure. Trm show little propensity to reenter circulation, and if they do, they have no directed pressure to establish themselves in other sites. This has best been shown in experiments where two mice – one antigen experienced and the other naïve – are surgically connected so that they share blood circulation. In this setting, only the antigen-experienced animal retains local Trm-mediated responses to rechallenge with the infectious agent (25, 117, 118). In the absence of further stimulation, Trm may traffic as far as the tissue draining lymph node, in a slower event over the course of weeks to months following their initial local antigen exposure (119). These cells retain residency features in the lymph node, and this mechanism can ensure locoregional memory within both the tissue site and the draining lymphatics to provide rapid response to infection (119). The literature is divided on whether these cells can re-enter circulation on rechallenge (27, 35), with some studies showing antigen challenge causes only local proliferation (35), other studies showing that rechallenge with a local infectious agent can cause the Trm to proliferate locally, enter the draining lymphatics and form conventional circulating memory populations (27). These data are impactful for understanding the Trm population in tumors. While cells with a Trm phenotype in tumors do not share TCR sequences with T cells in the draining lymphatics or the peripheral blood (38), it is possible that appropriate activation of these cells can cause recirculation. Similarly, it has been shown that circulating memory cells can become Trm following a subsequent local antigen challenge (120). Thus, in tumors where antigen is chronic, there may be a greater potential for turnover between resident and circulating tumor-specific T cells even though it is difficult to measure this with only steady state data. Yet, as discussed earlier, cells with Trm phenotypes in the tumor have unique clonotypes that are not readily detectable in the tumor-draining lymph node or peripheral circulation (38). These data suggest that despite chronic antigen presence, tumor-associated Trm are not measurably recirculating. As will be discussed later, Trm in the tumor express a range of exhaustion markers (38, 121) that are not typically observed on Trm in post-infection normal tissue. It is possible that by the time cancers become clinically evident, the reactivation potential of the tumor-infiltrating Trm is greatly reduced, and the cells that remain in the tumor have achieved a degree of balance between the antigen-presenting capacity of the cancer cells and their activation state.

### Stromal Versus Cancer Distribution of T Cells

**Figure 3:**
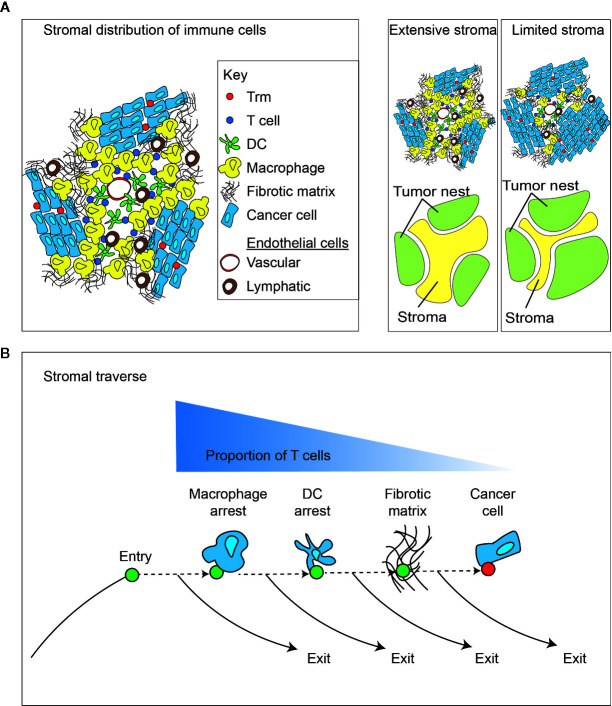
Impact of the tumor stroma and the stromal traverse on T cell dynamics. **(A)** The tumor is a non-homogenous structure that can be generally split into tumor and stroma. A diverse population of immune cells enrich in stroma away from direct cancer cell contact, and closer to vascular points of entry and lymphovascular points of exit. Different tumors can vary widely in the extent of tumor stroma. capacity for direct cancer cell cytotoxicity. **(B)** Following entry of lymphocytes into the tumor stroma *via* the vasculature, there are multiple stromal barriers that can cause T cell arrest and provide opportunities for a dominance of exit signals for continued recirculation through efferent lymphovasculature, rather than continuing through the stroma to meet cancer cells. More extensive stroma may increase the duration of traverse and decrease the likelihood of T cells meeting cancer cells.

The tumor is not a homogenous structure, and there are microenvironments within the broader tumor environment. One of the more critical distinctions is between the nests of cancer cells and the tumor stroma (**Figure 3**). The extent of tumor stroma varies considerably between individuals and between tumor pathologies. In addition, tumors can incorporate tertiary lymphoid structures, which are lymphocyte aggregates with varying levels of organization that can be found in cancer, and are similarly found in other scenarios where chronic inflammation disrupts the tissue architecture (122). In pancreatic cancer, the presence of these structures does not correlate with the overall tumor mutational burden (123), though the lymphoid structures are more likely where strong MHC-binding neoantigens are present. Tumors with tertiary lymphoid structures are likely to have more T cells infiltrating the tumor, but these T cells are less enriched for CD103^+^ cells (123), suggesting that the formation of tertiary lymphoid aggregates versus cancer-associated Trm occur through distinct mechanisms. Notably, the 12 chemokine gene signature used to predict T cell infiltration in tumors also predicts the presence of tertiary lymphoid structures in tumors (59, 60). However, it remains unclear whether there is direct movement between the lymphoid structures and the vicinity of the cancer cells. B cells in these structures can recognize tumor-associated antigens (124), so it is reasonable to infer that there are CD4 T cells with similar specificities in the lymphoid structures. However, thus far there is no direct evidence the tertiary lymphoid structures of tumors are significant sources of the tumor-specific effector CD8 T cells that participate in tumor control. Pancreatic cancer is a commonly mentioned example where it is often possible to detect extensive desmoplastic stroma punctuated with relatively small nests of cancer cells (**Figure 3**). The stroma can incorporate a diverse set of non-cancer cells and plays an important role in tumor growth and immune regulation. T cells are not uniformly distributed between the stroma and cancer cell nests – they are generally enriched in the stroma (49, 125, 126). Importantly, T cell subtypes are differentially distributed between stroma and cancer cell nests. For example, in breast cancer it has been demonstrated that cells with the Trm phenotype are enriched in cancer cell nests rather than the tumor stroma (126). By contrast, stem-like CD8 T cells expressing the Tcf1 marker were shown to be enriched in the immediate perivascular region (127). These stem-like cells have been shown to co-localize with APC in their stromal niche (128), suggesting that their interactions are more impacted by cross-presentation than by direct presentation by cancer cells. Considering features regulating recirculation, it is important to know that lymphatic density is highest in the tumor stroma surrounding cancer cell nests, driven by both cancer cell and stromal cell factors that guide lymphangiogenesis [reviewed in (129)]. Similarly, vascular endothelia are a defining feature of the tumor stroma, and the proximity of these entry and exit vessels and their separation from cancer cells means that recirculation can occur without T cells having an opportunity to directly contact the cancer cells.

The role of lymphatic endothelial cells in tumor immunity is multifaceted (129), but includes direct negative regulation of T cell activation as a result of inflammatory feedback (130). Since lymphatic endothelial cells mediate T cell exit from tissues, loss of lymphatic endothelial cells would be expected to decrease the ability of T cells to leave the tissue site. If ingress into the tumor is sustained and exit decreased, this should result in T cell accumulation. However, since as discussed above, rapidly recirculating T cells are more likely to be non-specific, this may not impact outcomes. Alternatively, since lymphatic endothelial cells can suppress T cells (130), more effective local immune responses might also be expected if there are fewer lymphatic endothelial cells in the tumor. Interestingly, contrary to these expectations tumors implanted into mice that lack functional lymphatic endothelial structures have fewer T cells infiltrating the tumor and reduced overall inflammation in the tumor (131). One possible explanation for this data is that impaired recirculation also results in impaired initial anti-tumor immunity, which will skew these results. As discussed earlier, to initiate new immune response dendritic cells must travel from the antigen site to draining lymph nodes *via* lymphatics to meet and stimulate naïve T cells. Loss of lymphatics might also mean loss of this initial anti-tumor immunity. Consistent with this, in viral models a lack of lymphatic endothelial structures results in impaired local control because of impaired initial immune activation in the draining lymph nodes (132). This initial immunity to tumor implantation is dependent on cross-presenting cDC1 and requires CD40 to generate both CD4 and CD8 T cell responses to tumor-associated antigens in the draining lymph node (133, 134). Using adoptive transfer to overcome this initial limitation in T cell responses, transferred tumor antigen-specific T cells have been shown to improve tumor control where tumors lack lymphatic endothelial cells (131), and pre-existing virus-specific T cells were equivalently capable of controlling a viral infection in the absence of lymphatic endothelial structures (132). These data suggest that lymphatic endothelial cells are required to prime new immune responses, but do not impair or suppress tumor immunity by existing tumor-specific T cells. Interestingly, increasing lymphangiogenesis in tumors increased their responsiveness to immunotherapies, and is associated with changes in the T cell populations that were recruited into the tumor immune environment (135). It is difficult to isolate the exact contribution of lymphatic structures due to the myriad of mechanisms by which they can interact with immune cells and the tumor stroma. However, given their role as an immunoregulatory component of the tumor stroma, the lymphovascular cells have a significant capacity to regulate the tumor immune environment and T cell recirculation (28).

The extracellular matrix represents an additional important limiting factor in T cell motility within the tumor. As discussed above, Salmon et al. demonstrated high T cell motility in the tumor stroma, and their comparative exclusion from tumor nests (49). Fibers of the extracellular matrix were shown to prevent direct T cell interactions with cancer cells (49). In such a setting the newly infiltrated T cells find it difficult to physically interact with the cognate antigen, but have an easy path to draining lymphatics which may comparatively promote their exit. For these newly-entered T cells the antigen presenting cells within the tumor stroma may play a significant role. Macrophages are prevalent in tumor stroma and are important in driving neoangiogenesis, lymphangiogenesis, and matrix remodeling for cancer cells growth (71, 129). Therefore, these cells are located amidst the key features regulating recirculation. Using live cell imaging, Peranzoni et al. demonstrated that tumor macrophages formed stable interactions with infiltrating CD8 T cells in the tumor stroma which reduced T cell motility (125). Depletion of macrophages using CSF1R inhibition restored T cell mobility and increased direct T cell interaction with cancer cells (125). Thus, macrophages in the tumor stroma may limit T cell mobility and thus limit functional interactions with cancer cells. Importantly, while macrophages can take up tumor antigens and present them to CD4 T cells *via* MHCII, they cannot cross-present antigen to CD8 T cells *via* MHCI. Dendritic cells are present in dramatically reduced proportions compared to other myeloid populations in the tumor stroma (136, 137), but they have the unique capacity to cross-present cell-associated antigens to infiltrating CD8 T cells (137, 138). Using cancer cells expressing fluorescent proteins, Englehardt et al. identified that dendritic cells closest to the cancer cells had the most phagocytosed cancer-cell material (139). They demonstrated that T cells closest to cancer cells exhibited reduced motility *in vivo*, and also that the T cells exhibited a more prolonged interaction with the dendritic cells that were close to the cancer cells compared to those dendritic cells that were further away and that had less phagocytosed cancer cell material (139). These data support the role for stromal dendritic cells in cross-presenting tumor associated antigens and this may increase T cell retention even where the T cells do not directly contact cancer cells. Interestingly, these dendritic cells were poorly able to support T cell proliferation unless first treated with innate adjuvants (139), suggesting that they maintain an immature phenotype and are unable to provide adequate co-stimulatory signals and cytokines to support T cell proliferation or effector function. These data suggest that those APC closest to the cancer cells have more abundant antigen for cross-presentation, but in progressively growing tumors these cells cannot sufficiently activate anti-tumor immunity for tumor control. Thus, they increase retention of tumor-specific clones, but do not necessarily result in tumor elimination. Tumor antigen-specific T cells have been described as being trapped in a dendritic cell network, restricting their access to cancer cells (140). This closely matches the observed state in snapshot views of tumors, where tumor-specific T cell clones are enriched but in poorly functional states permitting progressive tumor growth. Consistent with this, under steady state conditions tumor dendritic cells often have impaired functionality (136, 141). Dendritic cells in tumors can become poorly functional early in tumorigenesis (142, 143), and tumors with poorly functional dendritic cells are also poorly responsive to conventional therapies (136). These data indicate that tumor-infiltrating T cells must pass both physical barriers and intercepting APC that may drive local tolerance before they can even directly access cancer cells (**Figure 3B**). The role of intratumoral dendritic cells in regulating T cell control of tumors remains controversial, in part due to difficulties in distinguishing myeloid subtypes. While cross-presenting dendritic cells are required to initiate immune responses to tumor-associated antigens (133), they can be dispensable for tumor control by adoptively transferred T cells (144). This continued exit of matured and maturing dendritic cells makes it difficult to interpret the biology of dendritic cells in tumors at steady state, since by definition, the tumor resident dendritic cells should be immature since the mature cells have exited. However, features of the tumor environment that keep dendritic cells in the environment without permitting their maturation have the potential to generate a tolerogenic APC barrier. A wide range of immune interventions aim to provide signals that can drive dendritic cells maturation (145, 146), and many of these have shown efficacy in preclinical settings.

### Impact of Cancer Cell Antigen Presentation on Recirculation

Antigen presentation is a regulated process and can be dynamically upregulated in response to stimuli. For both mice and humans, non-MHC genes that are integral to antigen presentation on MHC-I are also contained within the Mhc region, including the genes for tapasin, TAP1, TAP2, and LMP7; however, in both humans and mice the gene for β2m is located on a separate chromosome. Expression of classical MHC-I elements is mediated by three major regulatory elements: enhancer A, IFN-stimulated response element (ISRE), and the SXY module. Within MHC-I promoters, enhancer A elements are bound by NF-κB/rel family members and ISRE elements are bound by interferon regulatory factors (IRFs) including IRF1; these transcription factors notably mediate transcription of MHC-I proteins downstream of IFNγ and TNFα stimulation (147–149). The SXY module, comprised of S/W, X1, X2 and Y boxes, binds a number of nuclear factors including RFX (comprised of RFX5, RFXAP, and RFXANK/B), CREB/ATF, and NF-Y, which require a transcriptional regulator, either the Class I Transactivator (CITA, or NLRC5) or the Class II Transactivator (CIITA), to coordinate enhanceosome assembly (147, 150, 151). NLRC5 is a dominant regulator of MHC-I in most cells (152), and expression of NLRC5 can be induced by IFNγ (153).

Downregulation of antigen presentation on MHC-I is a common immune evasion mechanism employed by tumors (154). While decreased antigen presentation is a common feature of cancer, total loss of MHC-I expression (for example *via* biallelic loss of B2M) is less common (155), likely due to selective pressure from natural killer cells whose cytotoxic function is inhibited by the presence of MHC-I. Antigen presentation on MHC molecules can be reduced without total ablation by epigenetic suppression or genetic loss of factors regulating MHC-I expression (e.g. NLRC5) (156–158), downregulation of molecules involved in peptide loading onto MHC-I (159–161), loss of specific HLA alleles (162), or suppression of cytokine-activated pathways for augmenting MHC-I expression (e.g. loss of IFNγR/IFNAR or downstream JAK/STAT molecules) (163–165).

Conventional cancer therapies have the potential to regulate MHC expression by cancer cells. Reits et al. demonstrated that MHC-I expression is increased after radiation due to increased availability of intracellular peptides available for loading (166). Cancer cell irradiation can also activate the cGAS/STING pathway, triggering extracellular release of type I IFN (167). Ligation of IFNAR with type I IFN triggers downstream signaling *via* STAT1, STAT2 and IRF9 and leads to transcription of interferon-stimulated genes including MHC-I related proteins (168, 169); resulting in IFN-dependent upregulation of MHC-I by cancer cells (170). Similar mechanisms have been proposed for chemotherapy-induced activation of the STING-IFN pathway (171, 172). In addition, radiation therapy can upregulate NLRC5 independently of STING and IFN activation (173), potentially *via* distinct DNA-damage detection mechanisms. This suggests that cancer cell MHC expression can be regulated *via* an array of conventional approaches to alter lymphocyte dynamics in the tumor environment.

**Figure 4:**
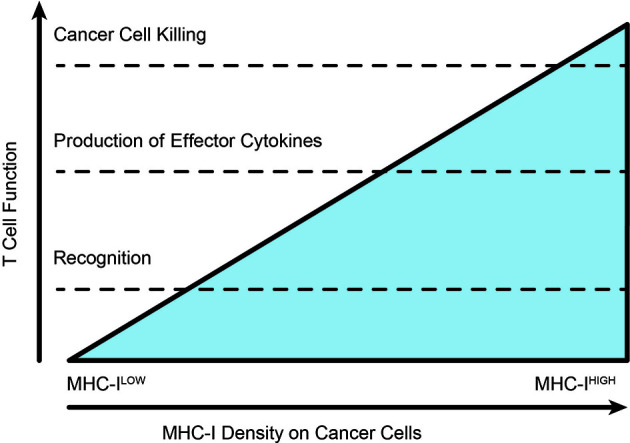
Varying thresholds for T cell function according to extent of TCR-MHC interactions. As the extent of cognate antigen increases on a target, whether by improved antigen expression or increased antigen processing and presentation, the intensity of T cell recognition allows the T cell to pass through various thresholds of response. The position of these thresholds can be altered by checkpoint blockade or costimulatory agonists. T cells in established tumors are typified by phenotypic modification without successful cytotoxic elimination of the cancer cells.

Significantly, alteration of cognate MHC-peptide expression on target cell surfaces can affect the magnitude and efficacy of CD8+ T cell responses (174). Differential responses of CD8+ T cells to varying MHC-peptide concentration have been observed, where increased epitope density corresponds with greater responsiveness to IL-2, enhanced proliferation and increased cytotoxic function including cytokine production (174–176). This phenomenon is better understood in naïve T cells, where high levels of antigen presentation in combination with costimulation and integrin stabilization are required to generate a stable immunological synapse and to cross an activation threshold of TCR signaling (174, 177). In activated T cells, MHC-peptide:TCR interactions at the synapse are much shorter: a single MHC-peptide complex can serially engage with rapidly internalizing TCRs and a CD8+ T cell can exert cytotoxic functions after engaging with as few as 1-3 MHC-peptide complexes per target cell (178, 179). It is clear that higher concentrations of MHC-peptide can engage more TCRs and it has been proposed that serial engagement of the TCR allows increased stability and enhanced signaling within the TCR/MHC-peptide/CD8 molecular complex (176). Functionally, downregulation of MHC-I induced by viral infection can significantly attenuate the ability of CD8+ T cells to kill infected targets (180). Interestingly, expression of the early activation marker CD69 appears to be independent of epitope density (181), and similarly CD69 and PD1 can be induced in the tumor environment independent of cognate antigen (182). Different densities of MHC-peptide can activate different thresholds in T cells for expression of early activation markers, cytolytic degranulation, versus cytotoxic cytokine release (183) (**Figure 4**). As discussed earlier, we have not explicitly examined the effect of local proliferation on T cell accumulation in tumors, instead grouping that as a retention mechanism. However, the degree of antigen presentation directly impacts the threshold for T cell proliferation in addition to cytotoxic activity. Since antigen presentation below a threshold can eliminate T cell responses, total loss of MHC-I is not necessary for resistance to T cells, and can result in various stages of activation without functional cancer cell cytotoxicity. This can explain why baseline tumor downregulation of antigen presentation *via* MHC-I results in checkpoint blockade resistance in human patients (164, 184), and presents a role for conventional therapies to increase T cell recognition and killing of cancer cells. In addition, antigen transfer that occurs as a result of cancer cell death driven by chemotherapy been shown to increase T cell infiltration into tumors, and increase T cell interactions with dendritic cells in the tumor (140). Similar mechanisms of antigen transfer to antigen presenting cells occur following radiation therapy (66), and have the potential to further manipulate the kinetics of lymphocyte movement through tumors by altering thresholds for cross-presentation in addition to direct presentation. Finally, T cell interactions that result in successful signalling and cytokine production can result in IFNg secretion and consequently increased antigen presentation in the vicinity of the T cell. While this may be tempered by simultaneous upregulation of negative regulation *via* PDL1, an initial cognate interaction by T cells can start a positive feedback loop in the microenvironment that can permit the T cell to pass critical activation thresholds that can result in cytotoxicity. For these reasons, manipulating the threshold for T cell activation in the tumor environment has the potential to dramatically alter tumor control.

### Effect of Checkpoint Regulators on Retention

As described above, while T cell entry into tumor is antigen-independent, retention in the tumor results from antigen-specific interactions with antigen-presenting cells or the cancer cells themselves. Immunotherapy has the potential to increase T cell surveillance of tumors through either mechanism – by increasing the recruitment or increasing their retention. However, to focus our efforts on antigen-specific cells it may be more useful to understand the mechanisms that increase the retention of this subpopulation in the tumor. As discussed earlier, we consider local proliferation to be a feature of retention, resulting in increased numbers of antigen-specific T cells in the site without changes in recruitment. In addition to the direct TCR interaction with cognate MHC-peptide on target cells, there are an array of costimulatory and coinhibitory signals that regulate that interaction (185). These are the targets of most of the immunotherapies currently being tested in clinical studies, and may function by regulating antigen-specific T cell retention and access to cancer cells, as well their function in the tumor, including local proliferation. The effect of checkpoint inhibition on TCR diversity and clonality has recently been reviewed (186), and this is an area of rapid research advancement. For example, Zhang et al. demonstrated that patients exhibiting a major pathological response to PD1 inhibition showed increased sharing of highly expanded clones between the tumor, peripheral blood, and non-tumor tissue (108). These data suggest that PD1 signals ordinarily limit T cell recirculation, or that the poorly responsive tumors in particular have limited active recirculation. Using live cell imaging it was shown that PD1 blockade increased the duration of T cell interactions with cancer cells (125), slowing their overall motility in the tumor environment. However, recent evidence suggests that PD1 blockade may increase the infiltration and function of a new clonally expanded population in the tumor, rather than restoring function of the existing exhausted cells (41). Single cell RNASeq plus TCR clonotype analysis of basal cell carcinoma before and after anti-PD1 therapy identified that T cells with shared TCR clones also tended to share a phenotype and that this phenotype was preserved following treatment (41), with exhausted clones remaining exhausted. Two thirds of the clones that expanded on treatment were new to the tumor, and included newly exhausted clones that made up the majority of exhausted cells in the treated tumor (41). Interestingly, this same precursor-like population is responsible for the proliferative expansion in T cells following PD1 blockade in viral models (187), suggesting that in both cases the response is dependent on expansion, recruitment and accumulation of new cells independent of the pre-existing clonally enriched, but exhausted T cells. Thus, in both viral infection models and tumor models, it has become clear that PD1 blockade is unable to restore the function of exhausted T cells and drive their conversion into memory and effector populations (188, 189). Rather, a distinct population current described as ‘progenitor exhausted cells’ that do not express the antigen-recognition and exhaustion marker CD39 expand following PD1 blockade and are more capable of generating effector function and tumor control (189). Siddiqui et al. demonstrated that these Tcf1+ progenitor-like cells were responsible for the majority of the proliferation following anti-PD1 therapy, and this expansion and tumor control could occur while new recruitment from the periphery was blocked using FTY720 (127). These data suggest that the progenitor-like clones may already be present in the tumor, and the apparent recruitment may be due to the frequency of these cells passing detection thresholds rather than recruitment from elsewhere. Since patients that have a pre-existing clonally expanded population of T cells expressing exhaustion markers is associated with improved outcome following PD1 blockade (47, 190), this suggests two major possibilities. Firstly, that the pre-existing population impacts the subsequent responses even if they are not the proliferative cells. Secondly, that the clonally expanded population in the tumor is evidence of a permissive environment in the cancer, which as discussed above might include effective antigen presentation and recruitment of cells into cancer cell nests to increase accumulation of tumor-specific clones. While it is currently difficult to break down these features, it is notable that clonal expansions of T cells following PD1 blockade occur in neighboring tissues as well as in the tumor (191). This suggests that the biology is not unique to the tumor but to applies to recirculating cells through other tissue sites.

Anti-CTLA4 therapy has been shown to increase T cell diversity in the peripheral blood of cancer patients (192, 193), though this was not necessarily reflected in positive phenotypic changes in the tumor (192). This data is consistent with expansion in functional specificities in the peripheral blood following anti-CTLA4 therapy (194), but little evidence of correlation between clonotypic changes in the peripheral blood versus the tumor following anti-CTLA4 therapy (195). Interestingly, in 4T1 mammary cancer models, anti-CTLA4 treatment increased motility of T cells in the tumor (196). Since this experimental design did not discriminate antigen-specific T cells it is possible that this increased motility reflects an increase in diverse T cell populations that are not tumor specific and so have increased overall motility in the tumor environment. Notably, adding radiation therapy to anti-CTLA4 therapy resulted in decreased overall motility in this model, and the change could be blocked with anti-MHCI antibodies, suggesting that cognate interactions are increased by the combination (196). TCRSeq in the same model shows that the combination of radiation and anti-CTLA4 increases the clonality of T cells in the tumor, and the proportion of antigen-specific T cells in the tumor, but no change in the distribution of TCR clones within the antigen specific population (197). This suggests that the tumor likely experiences increased infiltrates of clonal non-specific cells following anti-CTLA4 therapy. In patients, both anti-CTLA4 therapy and anti-PD1 therapy similarly increased T cell clonality in the tumor (190), but the extent of clonal expansion in the tumor pre-treatment was only predictive of outcome following anti-PD1 therapy (190). These data suggest the two agents have very different effects on T cell dynamics. By correlating patient responses to therapy with T cell populations in the blood, Wei et al. demonstrated that different T cell subpopulations in the peripheral blood correlated with response to single versus dual agent therapy (198). However, the frequency of these populations in the blood did not correlate well with their proportions in the tumor. Lau et al. demonstrated that combined PDL1 and CTLA4 blockade resulted in increased overall numbers of antigen-experienced T cells in murine tumors, and that these cells were more heterogeneously distributed than in untreated tumors (199). As observed in other models and discussed above, in these untreated murine tumor models T cells exhibited greater motility in the tumor periphery than in the tumor core (199). Following PDL1 blockade alone or combined PDL1 and CTLA4 blockade T cell motility was decreased in all regions of the tumor and overall infiltration was increased (199), consistent with increased cognate interactions in the tumor environment.

In contrast to checkpoint inhibitors like anti-PD1, costimulatory agonists like anti-OX40 (CD134) and anti-41BB (CD137) provide additional signals to T cells to overcome limited TCR stimulation, and can dramatically expand new populations of antigen-specific cells (200–202). This may occur because their antigen-specific interaction was below the necessary activation threshold as a lower affinity/avidity interaction, or because limited adjuvant signals have generated APC that are cross-presenting antigen but are not adequately providing costimulatory signals (203). In both cases, costimulation can generate a broader pool of antigen specific cells and also improve the quality of T cells as measured by memory formation and effector function (200–203). In the tumor, administration of anti-OX40 has been shown to increase the clonality of T cells in both the tumor and the spleen, suggesting that only some populations are being expanded by the therapy (204). Consistent with tolerogenic hypotheses limiting T cell responses in the tumor, T cells required anti-OX40 agonism to generate functional TCR signals in the tumor environment (182). In addition, higher affinity T cells in the tumor are more likely to express the costimulatory target OX40 (182), and the effects of anti-OX40 therapy was more pronounced on this tumor-infiltrating T cell population than those in the draining lymph nodes (182). These data suggest that OX40 costimulation specifically targets the existing antigen-specific T cell population that infiltrate but fail to cure tumors. Interestingly, the combination of anti-OX40 therapy and anti-PD1 therapy also enriches for T cells that receive high affinity TCR signals (204), as measured by activation of a Nur77-GFP reporter system (205). Thus, anti-OX40 therapy has been shown to remodel the tumor immune environment *via* activation of existing CD8 T cells that were previously functionally limited by the tumor immune environment (206, 207). To understand the response to agonist antibodies to 4-1BB, Weigelin et al. performed intravital imaging of tumors expressing ovalbumin as a model antigen to view tumor-specific responses of OT1 TCR transgenic T cells (208). As discussed above in other tumor models, these experiments demonstrated that OT1 T cells moved at slower speeds when tumors expressed their cognate antigen (208). The addition of agonist antibodies to 4-1BB (CD137) slowed the transit of the tumor-specific T cells, and increased the dwell time of T cells with target cells (208). These data demonstrate that as with PD1 blockade, costimulation can increase retention of T cells in the tumor resulting in T cell accumulation.

The consequence of manipulating T cell interactions with their cognate targets *via* checkpoint blockade and costimulatory agents can therefore be viewed through overlapping mechanisms. Firstly, new cells that previously were able to stably interact with their target can be incorporated into the anti-tumor immune response by decreasing the activation threshold of the T cells. This may occur *via* removal of negative regulation in T cell activation (209), or through provision of costimulatory support (210). Again, these data are consistent with ongoing immune surveillance of tumors by T cells as part of baseline recirculation. Those clones that are newly able to interact with cancer cells, can arrest and accumulate when checkpoints or costimulation are regulated. Secondly, there is clonal expansion. While the data is limited at present, costimulatory agents appear to expand existing populations that had been limited in their function in the tumor environment (204), while checkpoint inhibition through anti-PD1 appears to permit a new population of T cells to participate in the anti-tumor immune response (41). As the data improves, we will obtain a better picture of how these therapies impact recirculation kinetics versus local function, and how these features explain their effects in tumors.

### Conclusions

As we have discussed, lymphocyte exclusion from the tumor environment predominantly revolves around recruitment and recirculation kinetics, but within those contexts the retention of antigen specific T cells and their ability to meet cancer cells are key. A tumor with a high throughput of T cells through high recruitment and high recirculation is very *dynamic*. Such a tumor has a great deal of potential for T cell-mediated control of cancer cells should cells of the appropriate specificity exist. When faced with a tumor that is not very dynamic, meaning that infiltration is poor, it will likely also lack the inflammatory signals that recruit and mature dendritic cells, so such a tumor may fail to generate cells specific for tumor antigens and also not recruit any that are expanded or provided through immunotherapy.

However, there is little reason to assume that a high recruitment tumor has a higher proportion of tumor antigen specific cells. A random assortment of T cells being recruited to the tumor stroma would not be expected to impact tumor growth and progression, so a high degree of *entropy* would have no advantage even in a highly infiltrated tumor. Moreover, cells that recirculate through an inflamed tumor stroma may minimally pass out of the stroma and meet the cancer cells to permit antigen-specific destruction. If T cells can be recruited to cancer cell nests, it may not be necessary to have a high recirculation rate to eventually result in an accumulation of cancer-specific cells amongst cancer cells. A wide array of data discussed above suggests that this is the most important feature – high pre-existing clonality, high Trm infiltrates, T cells infiltrated into tumor nests. That is, a low entropy tumor.

Currently, therapies that assist the tumor specific T cells complete their tasks, such as anti-PD1 are the most effective immunotherapy agents in the clinic. As discussed above, recent data suggests that PD1 blockade functions to recruit a new population to participate in tumor control rather than convert the function of terminally exhausted cells. In turn, this suggests that an ability to direct these new T cells to the tumor is essential for responses. Therapies that will help expand the existing population of tumor-specific T cells, such as anti-OX40 and anti-41BB have not yet shown sufficient promise for clinical approval despite their preclinical power. Understanding the critical issues of recruitment and retention of tumor-specific T cells to the tumor, as well as mechanisms that allow us to initiate new anti-tumor immune responses where they are currently lacking, will be key to success. To do this we will need to look carefully so that we can discriminate these responses from the constant recirculation of irrelevant T cells, and understand how these might interact to regulate site specific immune responses, to control the dynamic entropy of tumor lymphocytes.

## Author Contributions

TB, writing, editing. AA, writing, editing. LZ, writing, editing. MC, writing, editing. MG, writing. All authors contributed to the article and approved the submitted version.

## Funding

This work was supported by NCI R01CA182311, R01CA244142, and R01CA208644.

## Conflict of Interest

MG and MC receive research funding from Bristol Myers-Squibb, Jounce, and Mavupharma that is unrelated to the content of this manuscript.

The remaining authors declare that the research was conducted in the absence of any commercial or financial relationships that could be construed as a potential conflict of interest.
